# Thick-Filament-Based Regulation and the Determinants of Force Generation

**DOI:** 10.3390/biomedicines13030703

**Published:** 2025-03-13

**Authors:** Vivek P. Jani, Weikang Ma

**Affiliations:** 1 Department of Biomedical Engineering, The Johns Hopkins School of Medicine, Baltimore, MD 21205, USA; vjani1@jhmi.edu; 2 Division of Cardiology, Department of Medicine, Johns Hopkins University School of Medicine, Baltimore, MD 21205, USA; 3 Department of Biology, Illinois Institute of Technology, Chicago, IL 60016, USA; 4 Center for Synchrotron Radiation Research and Instrumentation, Illinois Institute of Technology, Chicago, IL 60016, USA

**Keywords:** thick filament regulation, muscle contractility, cardiomyopathy

## Abstract

**Background/Objectives**: Thick-filament-based regulation in muscle is generally conceived as processes that modulate the number of myosin heads capable of force generation. It has been generally assumed that biochemical and structural assays of myosin active and inactive states provide equivalent measures of myosin recruitment, but recent studies indicate that this may not always be the case. Here, we studied the steady-state and dynamic mechanical changes in skinned porcine myocardium before and after treatment with omecamtiv mecarbil (OM) or piperine to help decipher how the biochemical and structural states of myosin separately affect contractile force. **Methods**: Force–Ca^2+^ relationships were obtained from skinned cardiomyocytes isolated from porcine myocardium before and after exposure to 1 μM OM and 7 μM piperine. Crossbridge kinetics were acquired using a step response stretch activation protocol allowing myosin attachment and detachment rates to be calculated. **Results**: OM augmented calcium-activated force at submaximal calcium levels that can be attributed to increased thick filament recruitment, increases in calcium sensitivity, an increased duty ratio, and from decelerated crossbridge detachment resulting in slowed crossbridge cycling kinetics. Piperine, in contrast, was able to increase activated force at submaximal calcium levels without appreciably affecting crossbridge cycling kinetics. **Conclusions:** Our study supports the notion that thick filament activation is primarily a process of myosin recruitment that is not necessarily coupled with the chemo-cycling of crossbridges. These new insights into thick filament activation mechanisms will need to be considered in the design of sarcomere-based therapies for treatment of myopathies.

## 1. Introduction

The primary function of muscle is to generate force by cyclic interactions between myosin-containing thick filaments and actin-containing thin filaments. Regulation of muscle contraction is critical to fulfilling the body’s specific needs. The underlying mechanism of contractile regulation has long been regarded as primarily a calcium (Ca^2+^)-mediated thin-filament-based mechanism [1,2,3], which assumes that all myosin heads are free to bind to actin once actin binding sites are made available after calcium binds to the troponin–tropomyosin complex. This assumption, however, has recently been reported to be false, and it is now clear that both thick- and thin-filament-based regulatory mechanisms are required to fully activate the sarcomere [4,5,6].

In the context of thick-filament-based regulation, Spudich has proposed that the total force in a sarcomere is dependent, in part, on the number of functionally accessible heads, N_a_, defined as those capable of force generation [7,8]. Changes in N_a_ are considered to be an important regulator in determining muscle force. Dysregulation of N_a_ has been proposed as a contributor to the sustained hypo- and hyper-contractility found in various myopathies, which has been leveraged for the development of new therapies for several cardiomyopathies [9,10,11,12]. Details of the mechanisms determining N_a_, however, have been elusive. The majority of myosin heads in resting muscle appear to be sequestered into a quasi-helically ordered state close to the thick filament backbone [13,14]. It is generally assumed that these structurally sequestered heads are in the super relaxed (SRX) state, defined by its low ATPase activity, in contrast to the disordered relaxed state (DRX) characterized by an ATP consumption rate approximately an order of magnitude faster [15,16,17]. It has also generally been assumed that these OFF-state heads are unavailable to bind actin and that the number of myosin heads in disordered states, whether biochemically or structurally defined, determine N_a_ and thus contractile force. There is substantial evidence to support this notion. For example, dATP has been shown to cause OFF-to-ON structural transitions [18,19] while promoting more myosin heads from the SRX to the DRX state [20] associated with increased mechanical output. On the other hand, the recently FDA-approved drug mavacamten can reduce myocardial contractile force by stabilizing both the structural OFF and biochemical SRX states [21,22,23,24].

Our previous studies, however, have shown that that the biochemical SRX/DRX and structural OFF/ON transitions do not necessarily occur in lockstep and can be decoupled. This was demonstrated as treatment of permeabilized porcine muscle with omecamtiv mecarbil (OM) or piperine increased the proportion of structurally ON heads without altering ATPase activity [25]. This raises serious questions as to what processes determine N_a_. The fact that OM and piperine decouple the biochemical and structural aspects of thick filament regulation makes them useful tool compounds for deciphering the respective roles of biochemical and structural transitions in modulating force generation and relaxation in muscle. Additionally, the large body of biochemical and clinical data available from OM makes it a valuable tool compound as a control to study other myosin activators that might be of clinical relevance. OM has been shown to not only recruit myosin heads but also modulate crossbridge chemo-cycling. Specifically, it has been shown that OM inhibits organic phosphate release [26] thus prolonging the period that force producing strong-binding crossbridges are bound, leading to force augmentation. OM also inhibits the velocity of actin sliding in in vitro motility assays [27] and reduces the rate of tension development and relaxation in myocytes [28]. While not much has been studied regarding the mode of action of piperine, an unanswered question is whether piperine also modulates crossbridge chemo-cycling.

Here, we studied the steady-state and dynamic mechanical changes in skinned porcine myocardium before and after exposing them to OM and piperine to investigate what factors determine the number of functionally accessible heads. To isolate the effects of OM and piperine on myosin recruitment from potential downstream crossbridge chemo-cycling, OM and piperine were excluded from the activating solution. This ensures that any bound OM or piperine present during the treatment was washed off shortly after exposure to the activating solution, allowing for the study of myosin recruitment in isolation. We showed that OM augmented calcium-activated force at submaximal calcium levels (~pCa 6), by thick filament recruitment, an increase in calcium sensitivity, and an increased duty ratio, from decelerated crossbridge detachment resulting in slowed crossbridge cycling kinetics consistent with previous studies [27,28,29]. Piperine, in contrast, was able to increase activated force at submaximal calcium levels without appreciably affecting crossbridge cycling kinetics. Our results imply that contractile force is determined by both the degree of myosin head recruitment from the thick filaments and cross-bridge chemo-cycling kinetics, two processes that are not necessarily coupled. This further implies that perturbations that result in myosin head recruitment from the thick filaments do not necessarily result in increased contractile force and vice versa. These new insights into thick filament activation mechanisms can inform the rational design of therapeutic approaches for cardiomyopathies.

## 2. Materials and Methods

### 2.1. Single Cardiomyocyte Mechanics

Porcine hearts were purchased from Exemplar Genetics (Sioux Center, IA, USA). The tissues were obtained in compliance with protocols approved by the Institutional Animal Care and Use Committees of Exemplar Genetics and no live animals were directly involved in this study. As a result, no additional institutional approval at the Illinois Institute of Technology or the Johns Hopkins University is required. Cardiomyocytes (CMs) were prepared as described [30]. Briefly, frozen porcine myocardium was cut into 10–15 mg pieces and permeabilized on ice in skinning solution (isolation buffer: 5.55 mM Na_2_ATP, 7.11 M MgCl_2_, 2 mM EGTA, 108.01 mM KCl, 8.91 KOH, 10 mM Imidazol, 10 mM DTT + 0.3% Triton X-100, all reagents from Sigma Aldrich, Burlington, MA, USA) with protease (Protease Inhibitor Cocktail, Sigma Aldrich, Burlington, MA, USA) and phosphatase (PhosStop, Roche purchased from Sigma Aldrich, Burlington, MA, USA) inhibitors. The tissue pieces were homogenized with low-speed pulverization, skinned for 20 min at 4 °C, and washed with isolation buffer to remove Triton. CMs were affixed to a force and length transducer using an ultraviolet-activated adhesive, and the sarcomere length was set to 2.1 μm as assessed by a high-speed video sarcomere length system (Aurora Scientific Inc., Aurora, ON, Canada). Force–Ca^2+^ relationships were obtained by subjecting the CMs to increasing Ca^2+^ concentration (from 0.0 to 46.8 μM). Force was normalized to the cross-sectional area estimated as (π/4)ab, where a is the diameter of the myocyte from the camera and b is the short axis diameter approximated as 0.8 a, to obtain tension (mN/mm^2^). The resting tension at a 2.1 μm sarcomere length was subtracted from the total tension measured to obtain the Ca^2+^-activated tension for each Ca^2+^ concentration. Steady-state tension versus the log [Ca^2+^] plots (T-Ca^2+^ plots) were fit to the three-element Hill equation: T = T_max_ × [Ca]^nh^/(EC_50_nh + [Ca]^nh^), where T_max_ is the maximum calcium-activated tension, EC_50_ is the calcium sensitivity, and n_h_ is the Hill coefficient. Following the acquisition of the first tension–Ca^2+^ relationship, the CMs were incubated in the relaxing solution with 1 μM OM and 7 μM piperine for 10 min, and the tension–Ca^2+^ relation post-exposure was obtained by the same protocol. Note that OM and piperine were not present in the activating solutions.

### 2.2. Crossbridge Kinetics

**Figure 1:**
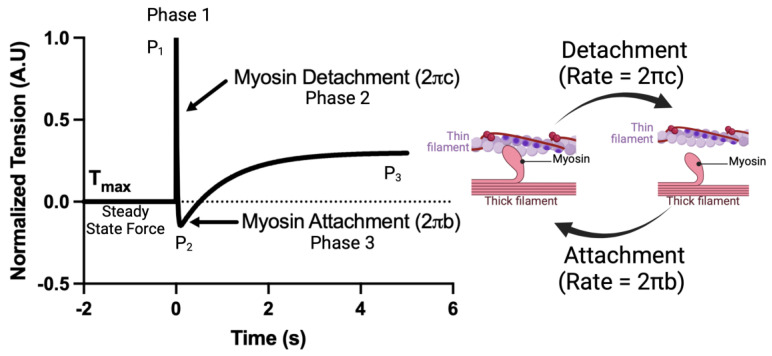
An example of the crossbridge kinetics protocol and a simplified interpretation of each fit coefficient.

Crossbridge kinetics, parameterized as 2πb and 2πc, were acquired using a step response stretch activation protocol as described previously [31]. CMs were prepared as described above. The CMs were set to a sarcomere length of 2.1 μm. The CMs were then moved to saturating calcium concentrations ([Ca^2+^] = 46.8 μM). After steady-state force was achieved, a small length step (2% cell length, <0.15% sarcomere length) was applied and force measured for 7 s at 2000 Hz. The resulting force tracing was normalized such that $\bar{F}$ = (F − F_max_)/F_step_, where $\bar{F}$ is the normalized force, F_max_ is the steady-state force, and F_step_ is the force immediately after the step response. The application of low strain perturbations to isolated cardiomyocytes is known to result in a three-phase tension waveform, consisting of the A process (complex modulus), B process (the stepwise cell lengthening disrupts the formed crossbridges, leading to a sudden drop in force (Figure 1); the force decay following cell lengthening can be used to calculate the crossbridge detachment rate), known as the C process (new crossbridges form soon after, accounting for force redevelopment (Figure 1); the force redevelopment trace can be used to estimate the crossbridge attachment rate). The resulting force trace takes the form. $\bar{F}\left(t\right)=P_{1}+P_{2}e^{-2\pi bt}-P_{3}e^{-2\pi ct}$, from which myosin attachment (2πb) and detachment (2πc) rates were acquired. A visual example of this protocol as well as a simplified interpretation of each fit coefficient is shown in Figure 1.

## 3. Results

### 3.1. Tension vs. Calcium Concentration Relationship Before and After OM and Piperine Treatment

**Figure 2:**
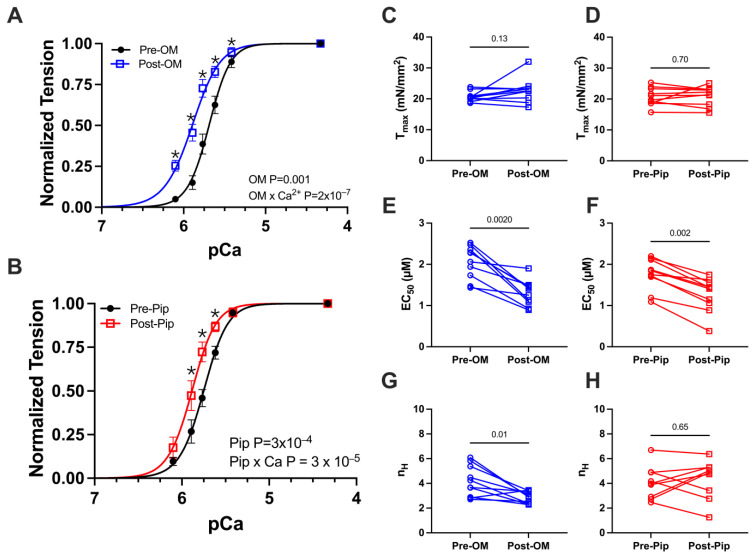
Tension vs. calcium concentration (pCa) relationship from permeabilized porcine CMs before and after OM and piperine (PIP) treatment. Active tension as a function of pCa before (black) and after OM (blue, **A**) and piperine (red, **B**) treatment. Tension under the maximally activated (Tmax) condition (**C**,**D**), the concentration of calcium at which half-maximal activation is achieved (EC 50) (**E**,**F**), and Hill coefficient (nH) of the tension calcium relationship (**G**,**H**) before (black) and after OM (blue) and piperine (red) treatment, respectively. The results are given as mean ± SEM, and *p* values from Hill fits (**A**,**B**) are calculated from 2-way ANOVA with Šídák’s multiple comparisons test and *p* values from column figures (**C**–**H**) are calculated from the Wilcoxon matched-pairs signed rank *t* test. * *p* < 0.05 for post hoc analysis with Šídák’s multiple comparisons test.

We first investigated active tension versus calcium concentration (pCa) relationships, with paired pre- and post-activator treatments. A classic sigmoidal tension–pCa relationship was observed in the control preparations (Figure 2A,B). Upon the addition of either OM or PIP, no change in maximum calcium-activated tension or T_max_ was observed (Figure 2C,D and Table 1). The addition of OM or PIP resulted in a leftward shift in the tension–pCa relationship, translating to differences in EC_50_, or the concentration of calcium at which half-maximal activation is achieved (Figure 2E,F and Table 1). There were also significant increases in tension at submaximal (systolic) calcium concentrations after treatment with OM (Figure 2A and Table 1) or PIP (Figure 2B and Table 1). Notably, the Hill coefficient was reduced with OM (Figure 2G and Table 1) but not PIP (Figure 2H and Table 1), the former consistent with prior reports.

### 3.2. Effects of OM and Piperine on Crossbridge Attachment and Detachment from Permeabilized Porcine CMs During Maximal Activation

**Figure 3:**
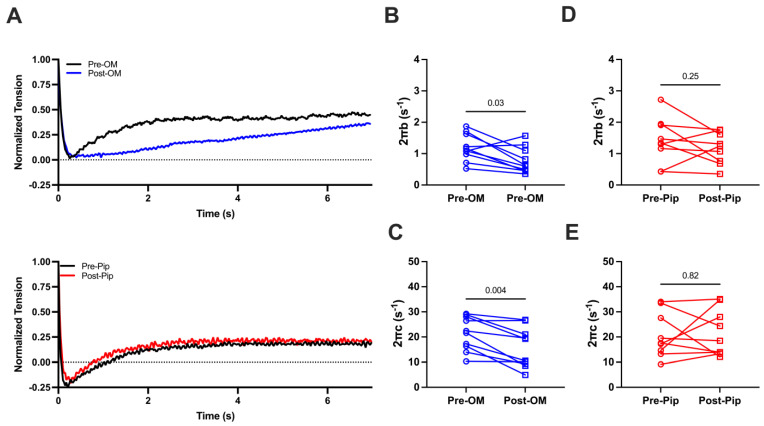
(**A**) The normalized force tracing after 2% CM lengthening before and after compound treatment. The crossbridge attachment rate (2πb, **B**) and crossbridge detachment rate (2πc, **C**) from CMs before and after OM treatment. The 2πb (**D**) and 2πc (**E**) from CMs before and after piperine (Pip) treatment. The results are given as mean ± SEM, and *p* values are calculated from the Wilcoxon matched-pairs signed rank t test.

We then investigated cross-bridge cycling kinetics by applying stepwise lengthening of the prepared CMs (2%) to disrupt crossbridge attachment and allow crossbridge reattachment to determine the crossbridge attachment rates (2πb) and detachment rates (2πc) before and after OM or PIP treatment during maximal activation (Figure 3A). Both 2πb values (1.20 ± 0.14 s^−1^ vs. 0.77 ± 0.13 s^−1^) and 2πc values (21.44 ± 2.14 s^−1^ vs. 15.67 ± 2/50 s^−1^) are significantly decreased pre- and post-OM treatment (Figure 3B,C), suggesting that OM slows both attachment and detachment kinetics. No significant changes in 2πb (1.41 ± 0.24 s^−1^ vs. 1.17 ± 0.17 s^−1^, Figure 3D) or 2πc (20.71 ± 2.98 s^−1^ vs. 21.55 ± 3.10 s^−1^, Figure 3E) pre- or post-PIP treatment were observed.

## 4. Discussion

*Thick filament activation and numbers of functionally accessible heads:* Force production requires both the recruitment of myosin and chemo-cycling of the crossbridges initiated by the calcium-dependent thin filament activation mechanism. While much is known about thin filament regulation, regulation of the thick filament has only recently been investigated. Thick filament activation involves the recruitment of myosin heads from sequestered ordered arrangements close to the thick filament backbone. Consistent with prior reports, our mechanical results showed that OM increases low calcium force and decreases cooperativity. Changes in T_max_, however, were less consistent in previous studies [29,32,33,34], and T_max_ was preserved with OM treatment in our study. Our mechanical data showed that piperine increases muscle steady-state force production at a submaximal level of activation without altering the crossbridge chemo-cycling. The advantage of our approach is that myosin recruitment can be studied independent of crossbridge cycling. These results, together with the fact that OM and piperine did not change the populations of myosin heads in the SRX and DRX states [25] indicate that force augmentation by OM and piperine observed here can be attributed to the increased level of myosin recruitment from the ordered OFF to the disordered ON states.

*Why does myosin head recruitment only change force production at submaximal activation levels?* We showed that OM and piperine during steady-state contraction induce a leftward shift in force pCa curves, indicating that cardiomyocytes are more sensitive to calcium in the presence of these compounds without significantly increasing T_max_. Similarly, other myosin activators such as EMD [30], dATP [19]**,** and danicamtiv [12] are also observed to have a more profound on force production at intermediate calcium levels than at saturating calcium levels. One possible mechanism for this phenomenon involves calcium-dependent thick filament activation. Thick filaments can be directly activated by calcium [35], and if thick filaments are partially turned ON by myosin activators, less calcium would be required to activate the muscle at intermediate levels of activation. The observation that these drugs do not change T_max_, however, could be because at full activation, the activating effect of either piperine or OM is offset by the effect of the saturating level of calcium. At these supraphysiologic calcium concentrations, the majority of myosin heads are already activated, which offsets any pharmacologic activation by these compounds. Other compounds, like dATP [19] and danicamtiv [12]**,** are observed to only slightly increase T_max_ as opposed to OM/PIP in this study. One possibility is that these compounds affect chemo-cycling properties by a different mechanism, which allows for an effect to be observed. The effect of myosin activators is larger in systolic HF patients with thick filament inhibition vs. non-failing patients and HF patients with a normal degree of thick filament activation [36], suggesting that Le Chatelier’s principle likely also contributes. It should be noted that Ca^2+^ binding or sensing sites on the thick filament have not been identified. We have previously speculated that one or more sarcomeric proteins such as the myosin regulatory light chain, myosin-binding protein C and titin, all known to bind to Ca^2+^, may be responsible for this Ca^2+^-mediated thick filament activation pathway [35,37]. These hypotheses should be tested in future experiments.

*Implications for sarcomere-targeted treatment of cardiomyopathies.* Myosin inhibitors have been proposed as a therapeutic strategy to curb the excessive contractility and impaired relaxation seen in Hypertrophic Cardiomyopathy (HCM). Indeed, this hypothesis has culminated in the FDA approval of mavacamten (Camzyos), a myosin-binding small molecule that stabilizes the myosin heads in the OFF state and decreases crossbridge chemo-cycling to treat obstructive HCM [11,38,39,40]. On the other hand, dilated cardiomyopathy and systolic HF patients are accompanied by depressed contractility, for which myosin activators have been proposed as a treatment. It has been shown that, at least in one cohort of right-ventricle heart failure patients, increases in the population of myosin heads in the OFF state may partially contribute to the underlying isometric tension in cardiomyocytes from these patients. One intriguing observation in this same study is that a population of myosin is neither calcium- nor length-sensitive, both of which are regarded as mechanisms for thick filament activation. While these myosin heads do share the same slow ATPase rate as the SRX state, they behave fundamentally differently from SRX myosin and may constitute a previously unrecognized immobilized relaxed (IRX) state, not recruitable by calcium, unlike heads in the SRX state [21,35,36,41,42]. It is likely that myosin in this pathological IRX state is less sensitive to other inotropic interventions, such as diastolic filling and β adrenergic stimulations, that contribute to the depressed cardiac reserve commonly seen in heart failure patients. Importantly, these IRX state myosins are readily recruited by myosin activators like dATP [36]**,** and pharmaceutically activating these IRX myosins could be a promising therapeutic strategy for these patients.

Additionally, whether a myosin-targeting compound is an activator or inhibitor is usually defined by its effects on contractile output. One may envisage that myosin activators work primarily by recruitment of myosin heads as supported by previous findings from dATP [19], EMD [30], and danicamtiv [12], while myosin inhibitors work by stabilizing the OFF state as supported by the evidence from compounds like mavacamten [11,43] and blebbistatin [44,45,46]. However, the helical ordering of myosin heads under resting conditions can be disrupted in various ways, and there are multiple examples of the decoupling of these two aspects in the literature. For instance, lowering temperature can disrupt the helical ordering of the myosin heads accompanied by a radial movement of the heads towards actin [47,48], but these nominally OFF-to-ON transitions in the myosin heads lead to decreased active force as myosin ATPase activity is reduced as is cross-bridge cycling. It has also been shown that N-benzyl-p-toluene sulfonamide (BTS) does not appreciably affect the resting myosin head configuration in intact skeletal muscle [44] but significantly inhibits muscle contractility, in this case by inhibiting the myosin ATPase [49]. Although further studies are required to uncover whether this decoupling occurs under physiological conditions and how it affects muscle performance, it is clear that for any compound that might target the SRX/DRX or the OFF/ON equilibrium as a therapeutic route for myopathies, one cannot assume they are necessarily coupled.

## 5. Conclusions

Our study supports the notion that thick filament activation is primarily a process of myosin recruitment and that it is not necessarily coupled with the chemo-cycling of crossbridges. The relative contribution of these two processes to the contractile output can, therefore, be independent. We propose that one cannot assume that a myosin inhibitor necessarily stabilizes the OFF state and that a myosin activator necessarily promotes more myosin in the ON state. Our findings provide a fresh look at the relationship in thick filament regulation and contractile output, which will be of great relevance in better designing sarcomere therapies aimed at reversing sarcomere-level dysfunction in myopathies.

## Figures and Tables

**Table 1:** The tension (mN/mm^2^) and calcium concentration (pCa) relationship before and after either omecamtiv mecarbil (OM) or piperine (PIP) treatment.

	Ctrl	OM	*p* Values	Ctrl	PIP	*p* Values
pCa 8	0	0	ns	0	0	ns
pCa 6.10	1.04 ± 0.26	5.73 ± 0.78	****	1.92 ± 0.45	3.68 ± 1.28	ns
pCa 5.89	3.18 ± 1.93	10.32 ± 1.26	****	5.34 ± 1.23	10.35 ± 1.95	****
pCa 5.77	8.15 ±1.32	16.49 ± 1.44	****	9.50 ± 1.03	15.41 ± 1.17	****
pCa 5.62	13.17 ± 1.21	18.58 ± 0.89	****	15.15 ± 1.28	18.57 ± 0.76	**
pCa 5.42	18.58 ± 0.73	21.52 ± 1.39	*	19.76 ± 0.98	20.40 ± 0.98	ns
pCa 4.33	20.98 ± 0.54	22.71 ± 1.20	ns	20.85 ± 0.93	21.62 ± 1.16	ns
T_max_	20.70 ± 0.52	22.70 ± 1.25	ns	20.72 ± 0.92	20.98 ± 0.97	ns
EC50 (μM)	2.06 ± 0.13	1.34 ± 0.31	**	1.76 ± 0.12	1.27 ± 0.13	**
nh	4.18 ± 0.40	2.88 ± 0.15	*	4.07 ± 0.44	4.34 ± 0.52	ns

Values are given as mean ± SEM. The tension and pCa relationships were fitted with a Hill equation, and the *p* values at each pCa value were calculated from 2-way ANOVA with Šídák’s multiple comparisons test. The *p* values for T_max_, EC50, and nh were calculated from the Wilcoxon matched-pairs signed rank *t* test. *n* = 10. * *p* < 0.05, ** *p* < 0.005, **** *p* < 0.0001.

## Data Availability

The original contributions presented in this study are included in the article. Further inquiries can be directed to the corresponding author.
